# Creativity as a Positive Factor in the Adolescence Stage: Relations with Academic Performance, Stress and Self-Esteem

**DOI:** 10.3390/bs13120997

**Published:** 2023-12-04

**Authors:** Alba González Moreno, María del Mar Molero Jurado

**Affiliations:** Department of Psychology, University of Almería, 04120 Almería, Spain

**Keywords:** creativity, academic performance, stress, self-esteem, adolescence

## Abstract

Creativity is a construct that aids in conflict resolution. Through the development of creative skills in adolescence, young people can carry out a series of strategies to make decisions or respond to a problem. The possession of creative skills helps students’ personal wellbeing. The aim of this research is to analyze the relationships established between creativity and other individual variables such as academic performance, self-esteem and stress in adolescent students. The following descriptive cross-sectional study was carried out with a total sample of 743 adolescent students, between 14 and 19 years of age, from different educational centers in the province of Almería (Spain). The results obtained indicate a positive correlation between creativity and self-esteem and significant differences in the level of creativity among students who have repeated an academic year. Regression analyses indicate that both stress and creativity are two predictor variables of self-esteem. On the other hand, another of the findings obtained is that creativity acts as a mediating variable between self-esteem and repeating an academic year. It is discussed how creativity is a beneficial element in adolescence and, therefore, how its promotion can help the optimal development of adolescent students.

## 1. Introduction

Creativity is a concept that has several different perspectives to be understood, due to its application in multiple everyday activities [1]. This construct is understood as being the ability of people to create something new to satisfy their needs or solve a problem [2]. Other authors characterize creativity as something that does not conform to conventional thinking and societal norms [3]. Although there is variety in the way creativity is understood, researchers of this construct agree that most experts see that creativity development is based on a combination of two aspects: originality and usefulness [4]. There are several paradigms that deal with the development of creativity, such as the theoretical model of productive thinking [5]. This model is structured in three levels: the first one covers the knowledge and motivational elements that the subject possesses, the second one discusses the tools of creative thinking such as fluency and originality and, finally, the third level details how the relationship of the two previous ones results in conflict resolution or decision making. Authors such as Trefinger [6] explain creativity as a product developed from a series of strategies that people use to reason, make decisions, solve problems or give meaning to life, so they consider it necessary to examine creativity from the construct of productive thinking. This link between creativity and conflict resolution was also previously proposed by Osborn [7], with his creative problem-solving (CPS) model, which indicated how people apply six steps to respond to a problem: searching for chaos, identifying the facts, defining the problem, searching for ideas, searching for solutions and implementing the solution. Problem solving is one of the variables most studied by the existing literature in conjunction with creativity at the secondary education stage [8].

Adolescence is a time of change that implies the need for young people to have the necessary strategies to respond to the problems of their daily lives [9,10]. The development of creativity in adolescents is influenced by four supportive categories that help creativity progress: individual factors, such as the intrinsic motivation of the subjects, parental factors, such as family support, educational factors, such as flexible and open-ended activities with learning expectations and social contextual factors, such as peer interactions that encourage challenging ideas and seeing problems from different perspectives [11,12]. Creativity is not an innate ability but, through the right teaching practices, can be taught and developed [13,14]. Teaching practices have not changed much over the years, so classroom activities are not geared towards fostering creative thinking skills [15,16]. It is worth highlighting the importance of fostering a positive school climate to enhance adolescents’ personal well-being [17].

Although the scientific literature on creativity focused on the adolescent stage is scarce, there are studies that argue for the benefits of creative skills at this age [18]. Following these benefits, it is interesting to delve into the variables that are related to creativity and enhance proper adolescent development. In relation to academic performance, certain cognitive skills involved in creative production and problem solving, such as flexible thinking and reasoning, significantly predict students’ academic achievement [19]. Conversely, research shows how people with higher creativity scores can manage perceived stress in difficult situations, thus showing greater positive emotions and, therefore, leads to the conclusion that creativity is associated with greater personal well-being [20]. Thus, having a range of skills that enable effective conflict resolution is associated with lower stress and higher self-esteem and life satisfaction [21,22,23]. Some of the conflicts that can be resolved through these creativity skills include certain school problems such as cyberbullying, which is related to anxiety and depression in youth [24].

It has been found that one of the factors that can help subjects to cope with stress in adolescence is self-esteem, as self-esteem is a significant predictor of emotional and behavioral problems [25,26]. Regarding self-esteem and stress, students with a good academic performance score high on self-esteem and low on stress [27,28]. This relationship can also be found between academic achievement and self-esteem, as previous studies indicate a significant association between these two variables in adolescents [29]. One aspect to highlight is that well-being promotes positive behaviors, thus favoring other variables such as academic performance and self-esteem [30]. Regarding the differences found according to gender in adolescents, it is noted that boys show higher self-esteem compared to girls [31]. Additionally, other studies indicate that it is girls who report greater symptoms of stress than boys [32]. Regarding the gender-based differences in creative skills, it is noted that the differences between girls and boys are very small and inconsistent [33]. This positive relationship between creative adolescents and self-esteem is also shown in studies such as that by Kim and Chung [34], who argue that self-esteem is partially mediated by creativity.

Based on the above studies, it can be seen how creative skills can act as a positive element in adolescents’ self-esteem, academic performance and stress, thus enhancing students’ personal well-being and development.

### Aims of the Study and Hypotheses

The aim of this research is to analyze the relationships established between creativity and other individual variables, such as academic performance, self-esteem and stress, in adolescent students. Furthermore, it also aims to find out if there are differences according to the genders and academic performances of adolescent students in the variables of creativity, stress and self-esteem. In addition, we want to identify how creativity acts positively on self-esteem and academic performance. The hypotheses proposed in this study are as follows:

**H1:**  *Creativity is positively related to academic performance and self-esteem and negatively related to stress.*

**H2:**  *There are differences according to the genders and academic performances of adolescent students in the variables of creativity, stress and self-esteem.*

**H3:**  *One of the predictors of self-esteem in adolescence is creativity.*

**H4:**  *Creativity acts as a mediating variable in the relationship established between academic performance and self-esteem.*

## 2. Methodology

### 2.1. Study Design and Participants

The present quantitative study was conducted using a descriptive cross-sectional design, following the STrengthening the Reporting of OBservational studies in Epidemiology (STROBE) statement and its guidelines for cross-sectional studies [35].

The subjects who participated in this study were 743 secondary school students, 377 girls (50.7%) and 366 boys (49.3%). The ages of the participants ranged from 14 to 19 years old (*M* = 14.99; *SD* = 0.86). All of them were enrolled in different schools in the province of Almería (Spain), specifically in the third (50.7%) and fourth (49.1%) years of this educational stage. Most of the students were of Spanish origin (92.9%), although the sample was also characterized by other nationalities such as Colombian, Moroccan, Russian or Argentinian.

### 2.2. Instruments

The evaluation of the students was carried out through the creation of a booklet by the authors, with a set of instruments validated by other researchers. This booklet consisted of instruments to assess different variables, as well as an ad hoc questionnaire that provided sociodemographic information about the adolescents. The instruments used to assess the variables considered in this study were as follows:

- Creativity: The creativity of the students was measured by means of the Turtle Creativity Questionnaire [36]. This instrument was chosen because it provides an overall assessment of creativity through the answering of different items, and, therefore, makes it easier to assess students’ creativity. It is composed of 31 items with dichotomous responses, where students must answer according to whether they identify with the items or not (e.g., very imaginative storytelling). The level of internal consistency obtained was acceptable (*α* = 0.65).
- Academic performance: The academic performance of adolescents was examined through a series of questions incorporated in the ad hoc that addressed this issue. Two questions were asked, one related to failing subjects (e.g., have you failed any subject in the last year?) and the other focused on whether the students had repeated a year (e.g., have you ever repeated a year?). Both were answered with a dichotomous response.
- Self-esteem: To examine self-esteem, the original Rosenberg Self Esteem Scale (RSES) [37] was used, specifically its Spanish version, which had been adapted for adolescents [38]. This instrument is made up of 10 items, focused on the feelings that a person has about him/herself, which are answered using a Likert-type scale with four response options (1 = strongly disagree; 2 = disagree; 3 = agree; 4 = strongly agree). It presents an overall self-esteem score which, depending on the answers obtained, can range from low self-esteem (less than 25 points) to high self-esteem (30 to 40 points). The internal consistency obtained in this instrument was good (*α* = 0.83).
- Stress: The manifestations of stress in adolescents were assessed using the Spanish adaptation of the Student Stress Inventory Scale (SSI-SM) [39], developed by Escobar [40]. This instrument consists of a total of 22 items, which are answered on a five-point Likert scale (1 = not at all; 2 = rarely; 3 = sometimes; 4 = often; 5 = completely). The stress manifestations scale results in both a total stress score and a three-factor score: emotional manifestations (e.g., I feel irritated), physiological manifestations (e.g., I lose my voice or become hoarse) and behavioral manifestations (e.g., I act defensive towards others). A good internal consistency was obtained for the total scale (*α* = 0.89) and the Emotional Manifestations dimension (*α* = 0.87), acceptable for Physiological Manifestations (*α* = 0.71) and questionable for Behavioral Manifestations (*α* = 0.65).

### 2.3. Procedure

Different secondary schools in the province of Almería (Spain) were contacted for the collection of information. A total of six public schools agreed to participate in this research. Thus, a date was agreed between the school management and the authors to attend the data collection. All members of the educational community, such as teachers, families and students, were informed of the aims of the study and its anonymous and voluntary nature. Once consent had been obtained from the participants and their guardians, data collection began between February and June 2022. This research was approved by the Bioethics Committee on Human Research of the University of Almería (Spain) with reference UALBIO2021/025.

### 2.4. Data Analysis

The data collected have been recorded in the statistical analysis program SPSS, version 28 [41], with the intention of subsequent analysis. Using this statistical software, the reliability of the instruments used was calculated using Cronbach’s alpha coefficient. This coefficient is interpreted as follows: <0.5 is unacceptable, >0.5 is poor, >0.6 is questionable, >0.7 is acceptable, >0.8 is good and >0.9 is excellent [42].

A descriptive analysis was performed to provide information on the adolescents who participated in the study, as well as a Pearson bivariate correlation analysis, to determine whether there were significant relationships between the variables examined. The absolute values obtained were interpreted according to the categories proposed by Pearson [43]: no correlation between 0 and 0.10, a weak correlation between 0.10 and 0.29, a moderate correlation between 0.30 and 0.50 and, finally, a strong correlation between 0.50 and 1.00.

A Student’s *t*-test for independent samples was applied to find out the differences between sex and the variables examined in adolescent students. In addition, Cohen’s d [44] was calculated to estimate effect sizes in this test, which was interpreted by the following scores: <0.50 small; 0.50–0.80 medium; and ≥0.80 large. To test for differences in gender distribution and academic performance, a 2 × 2 contingency table was performed with the Chi-square statistic.

A stepwise multiple regression analysis was performed, with self-esteem as the dependent variable, to find out how this variable is associated with the predictors (creativity and stress). To understand the change in effect sizes, change statistics, regression coefficients and collinearity were considered. Statistical significance was set at a *p*-value of minus 0.05.

Finally, to carry out the simple mediation analyses, the predictor variable was, in each case, having failed a subject and repeating a year, respectively. In each case, the different manifestations of stress (emotional, physiological and behavioral) and creativity are introduced as potential mediators. Self-esteem was used as a response variable. For the computation of the mediation models, the PROCESS macro for SPSS [45] was used, applying the bootstrapping technique with estimated coefficients from 5000 bootstrap samples.

## 3. Results

### 3.1. Descriptive Analyses and Correlations

Firstly, a correlation analysis was carried out on the continuous variables, to check how they were related to each other. As shown in Table 1, the results obtained indicated that creativity correlated positively with self-esteem (*r* = 0.17; *p* ≤ 0.001), while stress correlated negatively with self-esteem (*r* = −0.57; *p* ≤ 0.001). The absence of a relationship between creativity and stress was highlighted.

### 3.2. Differences Found According to Sex and Academic Performance in the Variables Examined

The data referred to in Table 2 showed the existence of differences according to sex in different variables and/or dimensions of the constructs analyzed.

In terms of self-esteem, adolescent boys showed higher levels of general self-esteem than girls. Stress was examined according to its dimensions, with girls scoring higher in emotional and physiological manifestations, while boys scored higher in the dimension of behavioral manifestations. No significant gender differences were found for creativity. No significant effects were found in the distribution by sex for academic performance or for having failed a subject or repeated an academic year.

Table 3 shows the differences found according to academic performance, both in terms of failing subjects and repeating an academic year.

A higher self-esteem score was shown for adolescents who had not failed any subjects, compared to those who had failed. On the other hand, it was found that adolescents who had failed subjects showed higher levels in the stress dimensions of physiological and behavioral manifestations than those who did not fail.

In terms of academic performance in relation to repeating an academic year, it was observed that adolescents who had not repeated a year had a higher level of creativity than those who had repeated a year.

### 3.3. Predictive Value of Academic Performance, Stress and Creativity on Self-Esteem

To find out how self-esteem was associated with the other variables studied (creativity, academic performance and stress), a stepwise multiple linear regression analysis was performed. One aspect to highlight is that the relationships between these variables may not be linear, so we first performed a nonlinear analysis. However, the results obtained indicated that a linear regression was more appropriate and effective in this specific context.

Table 4 shows the strength of the associations found in the multiple regression analysis between the covariates and the dependent variable (self-esteem), according to the effect sizes (standardized *β*) and the variance explained (*R*-squared). This linear regression analysis was made up of two models of predictors of self-esteem as a dependent variable in adolescents. Model I accounted for 31% of the variance and was made up of the dependent variable and stress. Additionally, Model II explained 35% of the variance and was made up of the dependent variable, stress and creativity.

This last model (Model II) was the one that offered the greatest explanatory capacity (*R*^2^ = 0.35), thus explaining self-esteem by all the predictors. The stress predictor was the variable with the greatest explanatory weight in this model, although the impact of all the predictors (i.e., stress and creativity) was statistically significant (*p* < 0.001). Finally, we noted the absence of collinearity, by obtaining high scores in the tolerance indicators and low scores in Variance Inflation Factor (VIF).

Once the linear regression analysis had been carried out with the total sample, the next step was to examine this analysis with a focus on academic performance. Therefore, we analyzed the participants who had failed a subject in the last year and those who had repeated a year (Table 5).

Considering the students who had failed a subject in the last year, two models were established, and Model II’s dependent variable of self-esteem had an explanatory capacity of 32% (*R*^2^ = 0.32). Stress was the variable with the greatest weight in the explanation of the model, with stress and creativity being significant. The absence of collinearity was highlighted by obtaining high scores in the tolerance indicators and low scores in VIF.

As for students who had not failed any subject in the last academic year, Model II had the greatest explanatory capacity with 36% (*R*^2^ = 0.36). The stress variable had the greatest explanatory weight with the dependent variable (self-esteem), followed by creativity, although both variables were. By obtaining high and low levels of tolerance in VIF, the absence of collinearity was approved.

The results of the regression analysis, focused on students who had repeated a year, provided a single model with an explanatory capacity of 25% (*R*^2^ = 0.25). Stress was the only variable and, therefore, the one with the greatest weight in the explanation of the dependent variable (self-esteem). The absence of collinearity was established by obtaining high scores in tolerance and low scores in VIF.

With reference to adolescents who had not repeated a school year, two models were obtained, Model II being the one with the greatest explanatory capacity with 34% (*R*^2^ = 0.34). The predictor with the greatest weight in this model was stress, although significant scores were found for both stress and creativity. By obtaining high scores in tolerance and low scores in VIF, the absence of collinearity was established.

### 3.4. Mediation Models

Based on the results obtained, we considered the need to assess whether the different manifestations of stress and creativity may have been mediating the relationship established between failing a subject/repeating a course and the level of self-esteem of secondary school students. Thus, in both cases, the manifestations of stress (emotional (M1), physiological (M2) and behavioral (M3)), as well as creativity (M4), were introduced as possible mediators of self-esteem (Y).

**Figure 1:**
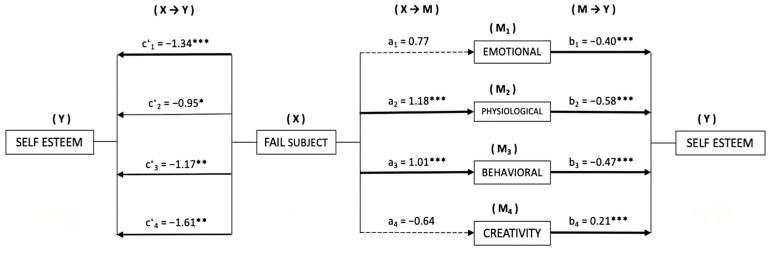
Mediation model of stress and creativity on the relationship between failing a subject and the level of self-Esteem. * *p* < 0.05; ** *p* < 0.01; *** *p* < 0.001.

In Figure 1, the mediation models are presented, taking subject suspension (X) as an independent variable.

First, a significant relationship was observed between failing a subject (X) and the manifestations of stress (M): physiological (*β* = 1.18, *p* < 0.001) and behavioral (*β* = 1.01, *p* < 0.001). The estimation of direct effects of X→Y showed the existence of significance of failing a subject on self-esteem (Y), in each of the models computed. On the other hand, regarding the estimation of the M→Y effects, we found significance on self-esteem (Y) in the different manifestations of stress (M): emotional (*β* = −0.40, *p* < 0.001), physiological (*β* = −0.58, *p* < 0.001) and behavioral (*β* = −0.47, *p* < 0.001); as well as in creativity (*β* = 0.21, *p* < 0.001).

With the analysis of indirect effects (X→M→Y), using the bootstrapping technique, significant values were obtained in two of the four models computed: physiological (*β* = −0.69, *SE* = 0.216, 95% CI (−1.149, −0.294)), and behavioral (*β* = −0.47, *SE* = 0.157, 95% CI (−0.826, −0.199)).

**Figure 2:**
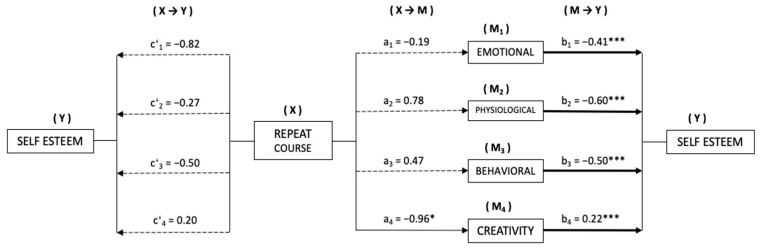
Mediation model of stress and creativity on the relationship between course repetition and level of Self-Esteem. * *p* < 0.05; *** *p* < 0.001.

In Figure 2, the mediation models are presented, taking course repetition (X) as the independent variable.

Firstly, a significant relationship was observed between repeating a course (X) and creativity (M) (*β* = −0.96, *p* < 0.05). However, no significant relationships were found with respect to the manifestations of stress (M): emotional (*β* = −0.19, *p* = 0.805), physiological (*β* = 0.78, *p* = 0.064), and behavioral (*β* = 0.47, *p* = 0.165). The results of the estimation of the direct effects (X→Y), meanwhile, revealed the absence of significant relationships of course repetition (X) on self-esteem (Y), in the computation of all models. As for the estimation of M→Y effects, we found significant effects on self-esteem in the three manifestations of stress (M): emotional (*β* = −0.41, *p* < 0.001), physiological (*β* = −0.60, *p* < 0.001), and behavioral (*β* = −0.50, *p* < 0.001); and also in creativity (*β* = 0.22, *p* < 0.001).

Finally, with the analysis of indirect effects (X→M→Y), using the *bootstrapping* technique, significant values were obtained only in the model that took creativity as a mediator (*β* = −0.21, *SE* = 0.115, 95% CI (−0.512, −0.040)).

## 4. Discussion

This study was oriented towards examining how creativity is a positive element in several variables immersed in adolescence, such as academic performance, self-esteem and stress.

As adolescence is a time of change, it is important for students to have the necessary skills and abilities to cope with problems in the best possible way [9,18]. Existing theoretical models in the previous literature indicate how creativity can provide original, useful and novel responses to conflicts [4,5,6,7]. Hypothesis 1 of the present study is accepted, as the results obtained indicated how creativity was positively correlated with self-esteem. This positive association between creativity and self-esteem is reflected in other studies that also show a positive association between creative adolescents and variables closely related to self-esteem, such as life satisfaction [20]. Thus, it can be noted that creative skills are related not only to higher life satisfaction and self-esteem, but also to a lower level of stress [21,23]. The scores obtained in the results revealed a negative correlation between stress and self-esteem, which meant that self-esteem acted as a moderator of stress [26]. These results suggest that the promotion of creativity could be an effective strategy to strengthen self-esteem in adolescents, thus contributing to their emotional well-being.

Hypothesis 2, that there would be differences according to gender and adolescent academic performance on the variables of creativity, self-esteem and stress, is also accepted to a greater extent. Regarding the differences found according to gender in adolescence, it is worth noting that boys seemed to have higher self-esteem and behavioral manifestations of stress. In contrast, girls had greater manifestations of emotional and physiological stress. These results are like those found in the previous literature, since girls are estimated to have lower self-esteem and higher stress compared to boys [31,32]. These data reinforce the idea that it is crucial to consider the differences between boys and girls when addressing adolescent well-being, just as it is necessary to adapt our strategies to deal with the specific forms of stress experienced by each gender. As for differences in academic performance, there were two approaches: failing subjects and repeating a year. Students who had failed a subject in the last academic year showed greater physiological and behavioral manifestations of stress, while those who had not failed had greater self-esteem and satisfaction with life. Conversely, students who had not repeated a year had a higher level of creativity compared to those who had repeated a year. Thus, we can highlight how there is a relationship between academic performance, emotional well-being and creativity. These data show how the interconnection between academic success, self-esteem and creative skills suggests that addressing each of these aspects in an integrated manner can have a significant positive impact on the overall well-being of adolescents.

To check the different elements that predict self-esteem in adolescence, a regression analysis was carried out. This analysis tested hypothesis 3, which stated that creativity is a predictor of self-esteem. These results showed how self-esteem was determined by stress and creativity. This analysis was carried out not only with the total sample, but also with the repeating and failing students. The results obtained indicated that the predictors of self-esteem in these students were mainly stress and creativity. This idea may be related to the fact that students with a good academic performance have higher self-esteem and a low level of stress [27,28]. The fact that stress and creativity remain the main predictors of self-esteem in these subgroups suggests that these variables may be crucial points of intervention to improve self-esteem, regardless of academic difficulties. The relationship between academic performance, self-esteem and creativity reinforces the idea that academic success not only contributes to higher self-esteem but may also influence adolescents’ creative ability. Thus, the relationship between creative output and academic achievement can be highlighted, as established in other studies [19]. The bidirectional relationship between creativity and academic performance suggests that fostering creativity could have positive benefits for both academics and the emotional well-being of adolescents.

Finally, Hypothesis 4 was answered. This hypothesis stated that creativity acts as a mediator between academic performance and self-esteem. The mediating role of different manifestations of stress and creativity on self-esteem was examined as a function of academic performance. It was found that for failing subjects, the dimensions of physiological and behavioral manifestations of stress did act as mediating variables of self-esteem. Furthermore, regarding repeating a year, creativity was the variable that acted as a mediator of self-esteem. These data are linked to the relationship between academic performance and self-esteem, and to the mediating nature of creativity versus self-esteem [29,34]. This analysis highlighted the interconnectedness of psychological and creative factors in the formation of adolescents’ self-image, especially in those with problems in their academic performance.

## 5. Conclusions

By way of conclusion, it is necessary to highlight how, through this research, it was possible to learn about the different correlations between the variables examined, their differences according to gender and the academic performance of the participants and, finally, how these variables predict and moderate the self-esteem of the adolescents. Some of the practical implications of this study include delving deeper into variables that are immersed in adolescence, such as creativity, self-esteem, stress and academic performance. However, it is important to recognize the inherent limitations of this research. The lack of specific scientific literature on creativity in the educational setting, especially at the adolescent stage, highlights the need for greater attention to this area. Furthermore, the measurement of creativity based on the perspective of the adolescents themselves and the evaluation of academic performance focused solely on repetition or passing courses could have introduced biases in the results. A crucial aspect to consider is the reliability of the creativity instrument, which was established at 0.65. Although this indicator suggests moderate consistency, it is essential to address this limitation when interpreting the findings. Future research could explore more robust and specific assessment alternatives. For future lines of research, it would be interesting to take a more specific measure of self-esteem, such as academic self-esteem, as this construct is too general and many factors beyond the academic sphere have an influence. In addition, it would be important to know what other variables influence adolescence and provide benefits in the development of young people, thus promoting their personal well-being.

## Figures and Tables

**Table 1:** Descriptives and correlation matrix between creativity, self-esteem and stress (*N* = 743).

	Creativity (CCT)	Self-Esteem (RSES)	Stress (SSI-SM)
Creativity (CTC)	-	-	-
Self-esteem (RSES)	0.17 ***	-	-
Stress (SSI-SM)	0.04	−0.57 ***	-
Mean	17.65	28.58	56.55
SD	4.27	5.91	14.65
Min.	1	11	23
Max	29	40	103

*** *p* < 0.001; CCT = Turtle Creativity Questionnaire; RSES = Rosenberg Self-Esteem Scale; SSI-SM = Stress Manifestation Scale.

**Table 2:** Differences according to sex in creativity, self-esteem and stress (girls *n* = 377; boys *n* = 366).

		Sex				*t*	*p*	*d*
		Boys		Girls				
		Mean	SD	Mean	SD			
Creativity (CCT)	Total creativity	17.57	4.52	17.72	4.01	−0.41	0.682	-
Self-esteem (RSES)	Total self-esteem	29.65	6.03	27.54	5.60	4.93 ***	<0.001	4.93
Stress (SSI-SM)	Emotional manifestations	28.27	8.43	33.40	8.03	−8.49 ***	<0.001	0.62
	Physiological manifestations	12.86	4.41	14.12	4.58	−3.80 ***	<0.001	0.28
	Behavioral manifestations	12.48	4.11	11.88	3.25	2.19 *	0.029	0.16

* *p* < 0.05; *** *p* < 0.001; CCT = Turtle Creativity Questionnaire; RSES = Rosenberg Self-Esteem Scale; SSI-SM = Stress Manifestation Scale.

**Table 3:** Differences according to academic performance in creativity, self-esteem and stress.

		Not Fail		Fail		*t*	*p*	*d*
		Mean	SD	Mean	SD			
Creativity (CCT)	Total creativity	18.03	4.30	17.38	4.06	1.70	0.088	-
Self-esteem (RSES)	Total self-esteem	29.37	6.06	27.71	5.70	3.54 ***	<0.001	0.26
Stress (SSI-SM)	Emotional manifestations	30.50	8.33	31.27	8.61	−1.14	0.252	
	Physiological manifestations	12.90	4.14	14.09	4.71	−3.37 ***	<0.001	0.25
	Behavioral manifestations	11.58	3.38	12.59	3.88	−3.48 ***	<0.001	0.26

*** *p* < 0.001; CCT = Turtle Creativity Questionnaire; RSES = Rosenberg Self-Esteem Scale; SSI-SM = Stress Manifestation Scale.

**Table 4:** Multiple linear regression model by steps (total sample; *N* = 743).

Variable	B	F	ß	*R* ^2^	T	*p*
Self-esteem						
**Model 1**						
Stress	−0.23	215.77	−0.56	0.31	−14.68	<0.001
**Model 2**						
Stress	−0.23	26.07	−0.57	0.35	−15.42	<0.001
Creativity	0.27		0.19		5.10	<0.001

**Table 5:** Multiple linear regression model by steps, according to academic performance.

Failing Subjects													
Fail							Not Fail						
Variable	B	F	ß	*R* ^2^	T	*p*	Variable	B	F	ß	*R* ^2^	T	*p*
Self-esteem							Self-esteem						
**Model 1**							**Model 1**						
Stress	−0.21	106.30	−0.54	0.30	−10.31	<0.001	Stress	−0.24	100.43	−0.54	0.29	−10.02	<0.001
**Model 2**							**Model 2**						
Stress	−0.22	9.95	−0.57	0.32	−10.83	<0.001	Stress	−0.24	18.22	−0.55	0.34	−10.48	<0.001
Creativity	0.23		0.16		3.15	0.002	Creativity	0.32		0.22		4.26	<0.001
**Repeat Course**													
**Repeat**							**Not Repeat**						
**Variable**	**B**	**F**	**ß**	***R* ^2^**	**T**	***p***	**Variable**	**B**	**F**	**ß**	***R* ^2^**	**T**	***p***
Self-esteem							Self-esteem						
**Model 1**							**Model 1**						
Stress	−0.19	37.50	−0.50	0.25	−6.12	<0.001	Stress	−0.24	234.81	−0.59	0.35	−15.32	<0.001
							**Model 2**						
							Stress	−0.24	31.54	−0.60	0.39	−16.18	<0.001
							Creativity	0.30		0.21		5.61	<0.001

## Data Availability

The data are available upon request to the corresponding author.
